# Janus kinase inhibitors effectively improve pain across different disease activity states in rheumatoid arthritis

**DOI:** 10.1007/s11739-023-03350-4

**Published:** 2023-07-27

**Authors:** Ludovico De Stefano, Emanuele Bozzalla Cassione, Francesca Bottazzi, Elena Marazzi, Francesco Maggiore, Valentina Morandi, Carlomaurizio Montecucco, Serena Bugatti

**Affiliations:** 1 Department of Internal Medicine and Therapeutics, Università di Pavia, Pavia, Italy; 2 Division of Rheumatology, Fondazione IRCCS Policlinico San Matteo, Pavia, Italy

**Keywords:** Janus kinase inhibitors, JAK, Rheumatoid arthritis, Pain, Difficult-to-treat, Refractory

## Abstract

Pain remains one of the most difficult-to-treat domains in patients with rheumatoid arthritis (RA). In clinical trials, the Janus kinase inhibitors (JAKis) have demonstrated good efficacy in pain relief. Aim of our study was to evaluate the real-life effectiveness of JAKis in improving pain in patients with RA in different states of baseline disease activity. A monocentric prospective cohort of 181 RA patients starting treatment with JAKis was studied. Pain was evaluated on a 0–100 mm visual analogue scale (VAS). Clinically meaningful improvements over 24 weeks were defined as follows: proportion of patients achieving ≥ 30%, ≥ 50%, and ≥ 70% pain relief, and remaining pain ≤ 20 or ≤ 10 mm. Results were analysed after stratification for baseline inflammatory activity; patients with swollen joints and C-reactive protein ≤ 1 at treatment start were considered pauci-inflammatory. Proportion of patients who achieved ≥ 30%, ≥ 50% and ≥ 70% pain improvement at 24 weeks was 61.4%, 49.3% and 32.9%. Furthermore, 40.6% and 28.5% of the patients achieved thresholds of remaining pain equivalent to mild pain or no/limited pain. Pain improvements were more evident in patients naive to previous biologics, although nearly 30% of multiple failures achieved VAS ≤ 20 mm. No significant differences were observed in relation to monotherapy. Pauci-inflammatory patients at treatment start achieved good outcomes, with 40.4% experiencing ≥ 70% pain improvement, and 35.7% VAS ≤ 10 mm. JAKis show efficacy in pain relief in real life. The improvement of painful symptoms also in those patients with limited objective inflammation may open new perspectives on the management of difficult-to-treat RA.

## Introduction

Outcomes of rheumatoid arthritis (RA) have dramatically improved over the past twenty years due to the recognition of the benefits of early diagnosis and treatment, as well as the increasing availability of a number of targeted disease-modifying antirheumatic drugs (DMARDs) [1]. Still, non-response to treatment remains a challenge in some patients, who experience persistently raised disease activity despite DMARD intensification and cycling [2, 3]. Relevantly, refractory joint and/or systemic inflammation impedes the achievement of acceptable clinical targets only in part. Raised disease activity scores can indeed also result from patient’s unsatisfaction and persistent pain despite the absence of apparent inflammation [4–6].

Joint tenderness and widespread pain uncoupled from perceivable inflammation are thought to arise from central sensitization and maladaptive pain processing mechanisms [7] and are considered mostly unamenable to immunosuppressive therapy. The exact pathophysiology of residual pain in RA remains, however, largely elusive and likely encompasses a multitude of mechanisms, some of which may still rely on inflammatory triggers. These include subclinical synovial inflammation and stromal-cell activation [8], antibody-mediated activation of afferent nerve fibres [9], and modulation of neuronal function and plasticity by cytokines and chemokines [10]. Supporting a neuro-immune-inflammatory component of pain in RA, imaging studies have revealed that neutralization of pro-inflammatory cytokines modifies brain connectivity before directly affecting joint inflammation [11].

The Janus kinase-signal transducer and activator of transcription (JAK/STAT) pathway directly and indirectly modulates a multitude of pro- and anti-inflammatory cytokines [12], and randomized clinical trials (RCTs) on JAK inhibitors (JAKis) in RA have demonstrated greater improvements in pain compared to other mechanisms of action despite similar efficacy on standard measures of inflammation [13–18]. The effect of JAKis on pain symptoms possibly appeared independent of clinical joint swelling in a single post hoc analysis of a RCT [15]. The additional independent effect of JAKis on pain, however, needs to be confirmed outside clinical trials. Aim of our study was to evaluate the real-life effectiveness of JAKis in improving pain in patients with RA in different states of baseline disease activity.

## Methods

### Patients

From the biologic (b)/targeted synthetic (ts) DMARD dedicated clinic of the Division of Rheumatology of the San Matteo University Hospital of Pavia, we selected all RA patients who began the first JAKi from time of licencing of tofacitinib in Italy (October 2018) to June 2022 and had 6 months follow-up available. Intra-class switches (i.e. subsequent JAKis after a first JAK) and different clinical indications (e.g. psoriatic arthritis) were excluded.

The study was conducted according to the declaration of Helsinki; all patients signed written informed consent before inclusion, and the local Ethics Committees approved the study protocol.

### Data collection and follow-up

At treatment start, demographic and clinical data were collected and included, among the others, disease duration, history of previous bDMARD exposure, and concomitant use of conventional synthetic (cs) DMARDs and glucocorticoids. Disease activity and impact were assessed at baseline and every 8 weeks thereafter through the following instruments: the tender and swollen joint count on 28 joints (TJC28, SJC28), the patient global assessment (PGA) and physician’s global assessment of disease activity on 0–100 mm visual analogue scales (VAS), VAS for general health (GH) and pain (0–100 mm), the erythrocyte sedimentation rate and C-reactive protein (CRP, in mg/dl) levels. Rheumatoid factor (RF) and anti-citrullinated protein antibodies (ACPA) were evaluated on electronic records before treatment start. Patients were classified as autoantibody-positive if RF and/or ACPA were above the reference cut-off values; autoantibody-negative in case of RF and ACPA both negative. Date and reason for drug withdrawal during the study period were recorded.

### Outcomes

After 8, 16 and 24 weeks of therapy, outcomes of interest included:

- Achievement of disease remission according to the 28-joints disease activity score calculated with CRP levels (DAS28-CRP < 2.6), the clinical disease activity index (CDAI < 2.8) and Boolean criteria (SJC28, TJC28, CRP and PGA all ≤ 1);
-  ≥ 30%, ≥ 50%, or ≥ 70% improvement of pain from baseline;
- Achievement of clinically meaningful thresholds of remaining pain: VAS pain ≤ 20 mm (mild pain) and ≤ 10 mm (no or limited pain) [15].

Outcomes were analysed in the overall population and after stratification for baseline inflammatory activity according to SJC28 and CRP at treatment start:

- SJC28 and CRP ≤ 1 = no or limited inflammation
- SJC28 > 1, CRP > 1, or both = overt inflammation

### Statistical analysis

Quantitative data were expressed as means and standard deviations (SD) and categorical data as frequencies. There was no imputation of missing data. Remission status over time was analysed using the Kaplan–Meier method, and the time-course of pain scores using repeated measures analysis of variance. Comparisons between groups were made by means of independent samples *t* test or chi statistics, as appropriate. All analyses were conducted using MedCalc® Version 12.7.0.0, and the significance level was set at 0.05.

## Results

### Baseline characteristics

The study population included 181 RA patients treated with a JAKi: tofacitinib *n* = 17, baricitinib *n* = 99 (8 receiving 2 mg/day), upadacitinib *n* = 47, filgotinib *n* = 18. The main demographic and clinical characteristic at treatment start are detailed in Table 1. Patients had mean [SD] disease duration of 9.4 [7.3] years and were autoantibody-positive in 75.1% of the cases. The JAKi was started as first b/tsDMARD in 38.7% of the cases, after failure of 1 bDMARD in 19.9%, and of ≥ 2 bDMARD in 41.4%; in this latter group, the mean (SD) number of previous bDMARDs was 3 (1.2). Seventy per cent of the patients received combination therapy with csDMARDs (mostly methotrexate), and a similar proportion was on glucocorticoids. Relevantly, approximatively one-third of the patients (*n* = 60, 33.1%) lacked significant synovial and systemic inflammation (SJC28 and CRP ≤ 1) despite being highly symptomatic (mean [SD] VAS pain 51.1 [25.6]). The characteristics of this apparent non-inflammatory group in comparison with the rest of the cohort are detailed in Table 1. Of note, only 2 out of the 60 non-inflammatory patients (3.3%) were in CDAI remission at treatment start, and none in Boolean remission.

**Table 1:** Baseline demographic and clinical characteristics of the study population

	Total cohort *n* = 181	SJC28 > 1, CRP > 1 or both *n* = 121	SJC28 and CRP ≤ 1 *n* = 60	*p*
Age, mean (SD)	56.9 (12.5)	58.2 (13.2)	54.4 (11.1)	0.06
Female gender, *n*. (%)	141 (77.9)	96 (79.3)	45 (75)	0.64
BMI, mean (SD)	25.2 (4.9)	25.8 (5.2)	23.9 (3.8)	**0.04**
Smoking, *n*. (%)	25 (13.8)	14 (11.6)	11 (18.3)	0.32
Fibromyalgia, *n*. (%)	24 (13.3)	10 (8.3)	12 (20)	**0.04**
Disease duration, mean (SD), years	9.4 (7.9)	9.1 (8.1)	9.7 (7.3)	0.63
RF and/or ACPA positive, *n*. (%)	136 (75.1)	93 (76.9)	43 (71.6)	0.55
Previous bDMARDs, n. (%)				
0	70 (38.7)	44 (36.4)	26 (43.3)	0.46
1	36 (19.9)	26 (21.5)	10 (16.7)	0.56
≥ 2	75 (41.4)	51 (42.1)	24 (40)	0.91
Concomitant csDMARDs, *n*. (%)				
None	55 (30.4)	35 (30.2)	20 (30.8)	0.93
MTX	73 (40.3)	46 (39.6)	27 (41.5)	0.93
MTX + other than MTX	36 (19.9)	24 (20.7)	12 (18.5)	0.87
Other than MTX	17 (9.4)	11 (9.5)	6 (9.2)	0.84
GC, *n*. (%)	126 (69.6)	89 (73.6)	37 (61.7)	0.14
GC dose, mean (SD)	6.1 (3.9)	6.8 (4.5)	4.7 (2.1)	**0.008**
DAS28-CRP, mean (SD)	3.61 (1.10)	3.98 (1.00)	2.83 (0.86)	** < 0.001**
DAS28-ESR, mean (SD)	4.01 (1.17)	4.36 (1.11)	3.18 (0.89)	** < 0.001**
CDAI, mean (SD)	16.1 (9.5)	19.4 (9.3)	9.8 (7.5)	** < 0.001**
SJC28, mean (SD)	2.8 (3)	4.1 (2.9)	0.4 (0.5)	** < 0.001**
TJC28, mean (SD)	4.6 (6.2)	5.7 (6.9)	2.7 (4.3)	**0.004**
PGA, mean (SD)	56.5 (28.5)	58.4 (29.4)	52.2 (25.5)	0.19
VAS pain, mean (SD)	55 (28.1)	56.2 (28.8)	51.1 (25.6)	0.27
CRP, mean (SD), mg/dl	1.12 (1.65)	1.52 (1.92)	0.35 (0.27)	** < 0.001**
ESR, mean (SD), mm/1 h	25.6 (21.7)	28.5 (20.9)	19.3 (20.3)	**0.01**

*ACPA* Anti-Citrullinated Protein Antibodies; *BMI* Body Mass Index; *b* Biologic; *cs* Conventional Synthetic; *CRP* C-Reactive Protein; *DAS28* 28-joints Disease Activity Score; *DMARDs* Disease-Modifying Anti-Rheumatic Drugs; *ESR* Erythrocyte Sedimentation Rate; *GC* Glucocorticoids; *MTX* Methotrexate; *PGA* Patient Global Assessment; *RF* Rheumatoid Factor; *SJC28* Swollen Joint Count on 28 joints; *TJC28* Tender Joint Count on 28 joints; *VAS* Visual Analogue Scale

Numbers in bold express statistical significance

### Clinical outcomes

**Fig. 1:**
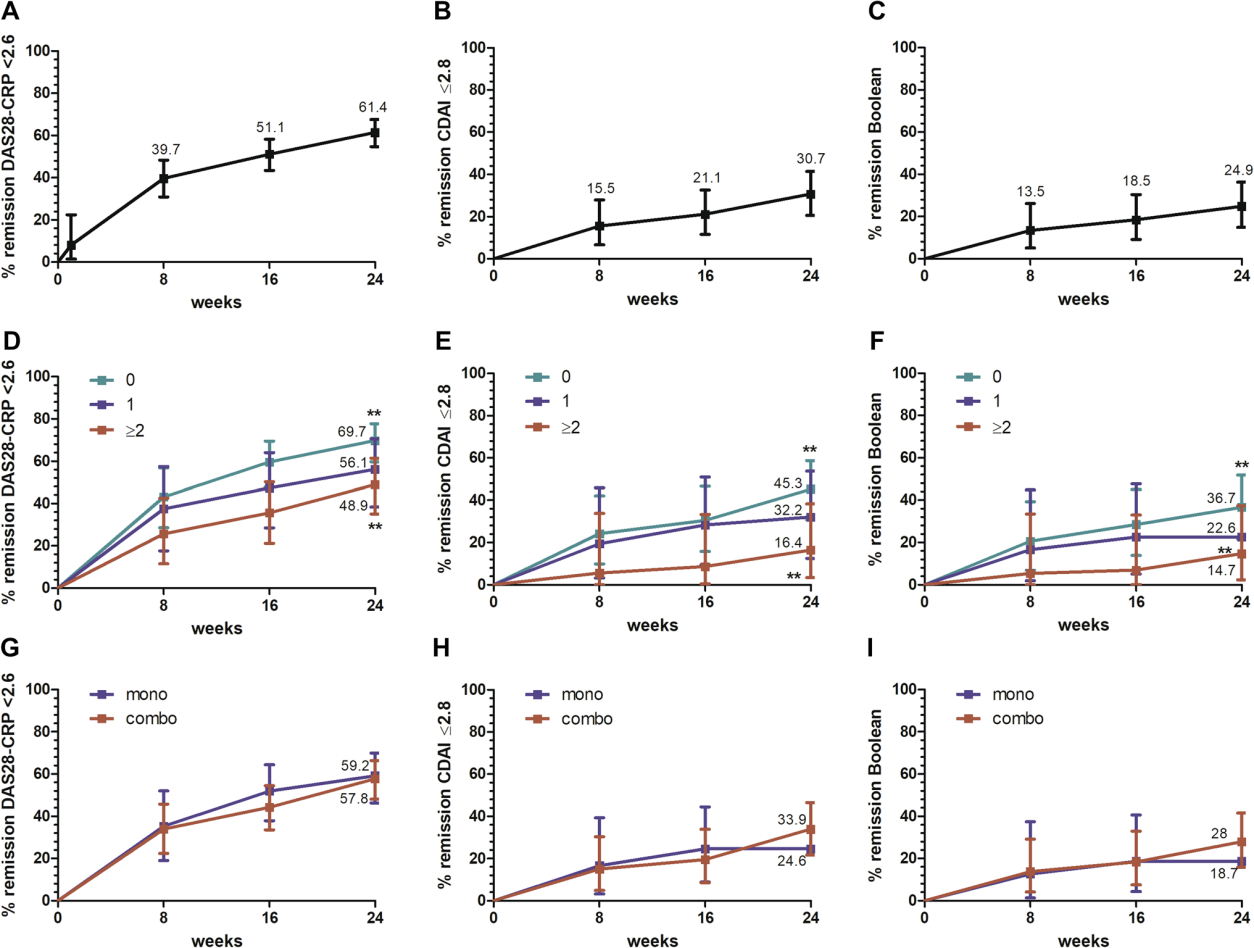
Percentage of patients achieving remission from baseline.Percentage of patients achieving remission according to the 28-joints disease activity score calculated with C-reactive protein levels (DAS28-CRP < 2.6) (**A**, **D**, **G**), clinical disease activity index (CDAI ≤ 2.8) (**B**, **E**, **H**) or Boolean criteria (**C**, **F**, **I**) are shown in the total cohort (**A**–**C**) and after stratification for treatment line (**D**–**F**) and monotherapy (**G**–**I**). For line of therapy, 0 = naive to biological disease-modifying anti-rheumatic drugs (bDMARD); 1 = 1 previous bDMARD; ≥ 2 =  ≥ 2 previous bDMARDs. **p < 0.001

During the 6 months of observation, 34 patients (18.8%) discontinued the JAKi after a mean (SD) of 16.4 (6.8) weeks due to clinical inefficacy (*n* = 19, 10.5%), adverse events (*n* = 14, 7.7%) or loss to follow-up (*n* = 1, 0.6%). Adverse events included non-serious infections (*n* = 9), herpes zoster (*n* = 1), neutropenia (*n* = 2), neurosensory hear loss (*n* = 1) and renal dysfunction (*n* = 1). Now major cardiovascular events, venous thromboembolism or cancer were observed. As shown in Fig. 1A–C, proportion of patients achieving remission according to DAS28-CRP, CDAI and Boolean criteria progressively increased over time, reaching 61.4%, 30.7% and 24.9%, respectively, at 24 weeks. As expected, remission was more common in bDMARD-naive patients, whilst no significant differences were observed in relation to concomitant use of csDMARDs (Fig. 1D–I). Among the patients with no or limited inflammation (SJC28 and CRP ≤ 1) at treatment start, CDAI remission was achieved in 37.9% of the cases, and Boolean remission in 32.8%.

### Pain outcomes

**Fig. 2:**
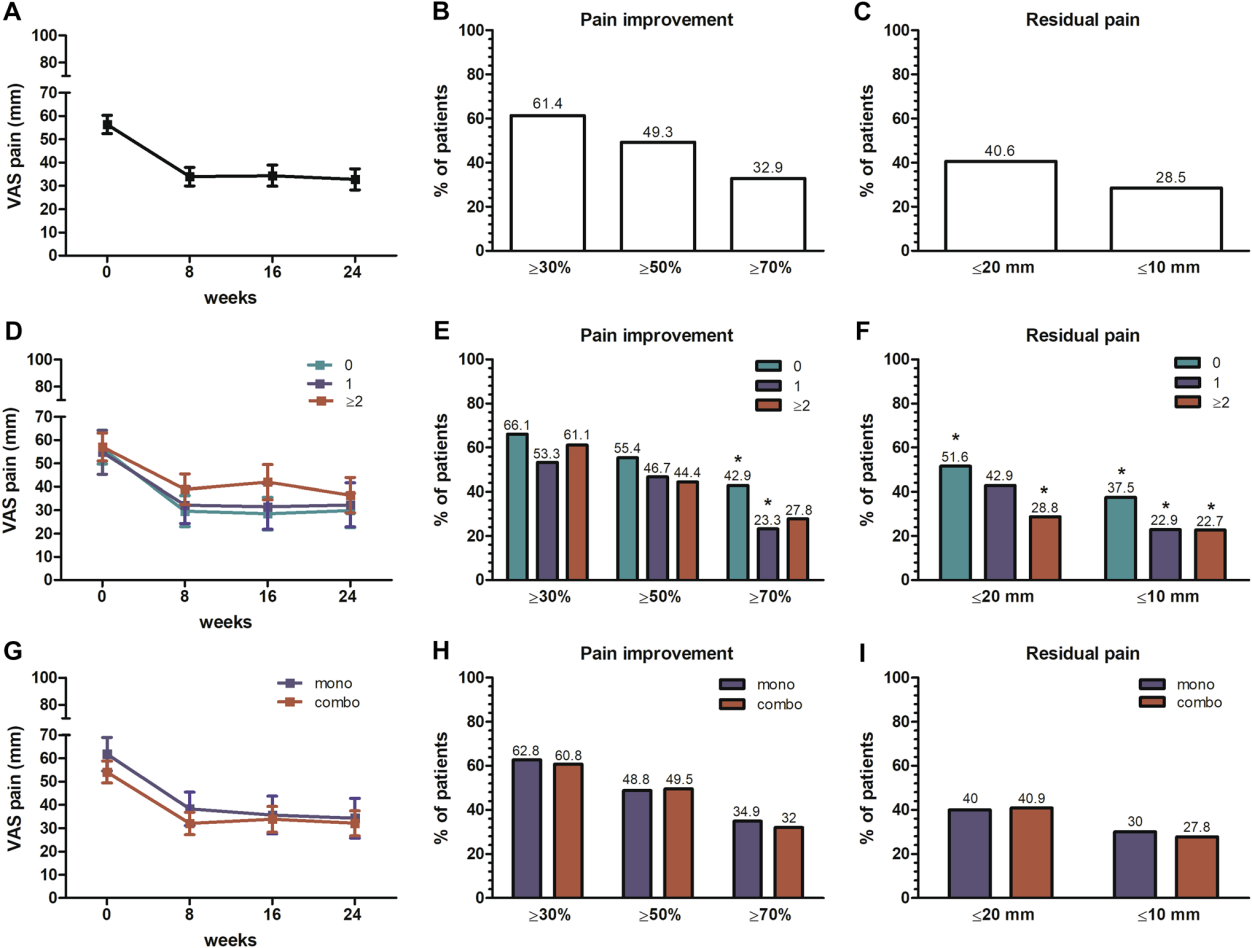
Pain improvement from baseline. Mean reduction in VAS pain from baseline (**A**, **D**, **G**), percentage of patients who achieved pain relief thresholds (**B**, **E**, **H**), and thresholds of negligible residual pain (**C**, **F**, **I**) are shown in the total cohort (**A**–**C**) and after stratification for treatment line (**D**–**F**) and monotherapy (**G**–**I**). For line of therapy, 0 = naive to biological disease-modifying anti-rheumatic drugs (bDMARD); 1 = 1 previous bDMARD; ≥ 2 =  ≥ 2 previous bDMARDs. *p < 0.01

In the total cohort, pain significantly improved from baseline from week 8 of a mean (95% CI) of  – 23.2 ( – 27.2 to – 19.2) mm, with no further major changes thereafter (Fig. 2A). Proportion of patients who achieved ≥ 30%, ≥ 50% and ≥ 70% improvement in VAS pain at their last available assessment was 61.4%, 49.3% and 32.9%, respectively (Fig. 2B). Furthermore, 40.6% and 28.5% of the patients achieved thresholds of remaining pain equivalent to mild pain (≤ 20 mm) or no or limited pain (≤ 10 mm) (Fig. 2C). As expected, pain improvements were more evident in bDMARD-naive patients, although nearly 30% of multiple failures (≥ 2 bDMARDs) still achieved mild pain (Fig. 2D–F). No significant differences were observed in relation to combination therapy with csDMARDs (Fig. 2G–I).

**Fig. 3:**
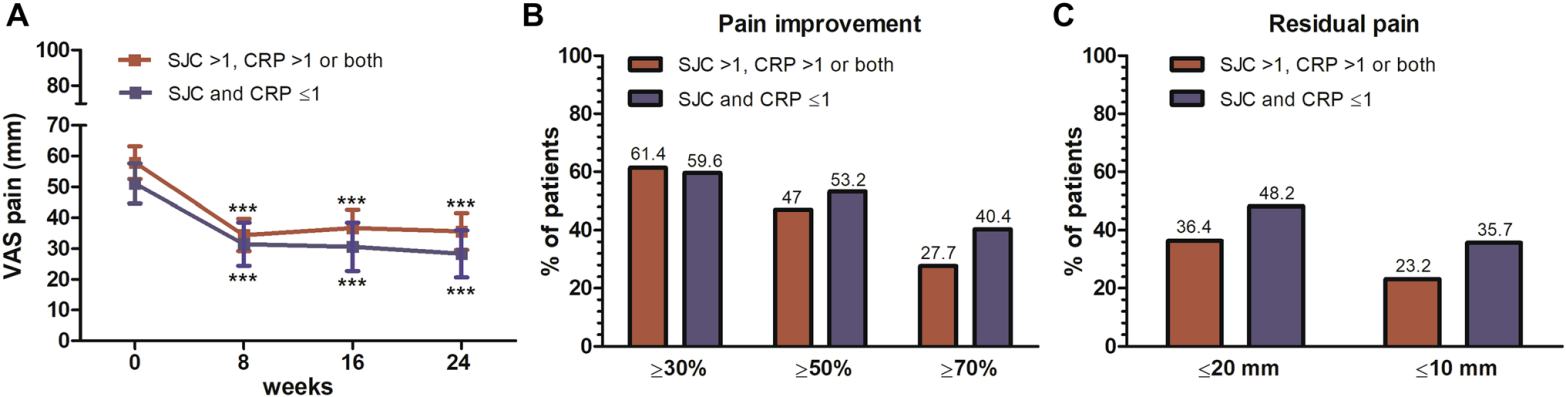
Pain improvement according to baseline disease activity. Mean reduction in VAS pain from baseline (**A**), percentage of patients who achieved pain relief thresholds (**B**), and thresholds of negligible residual pain (**C**) are shown in patients with baseline low levels of inflammation (swollen joint count, SJC, and C-reactive protein, CRP, both ≤ 1, in blue) and in patients with overt inflammatory disease activity (in red)

Relevantly, pain improvement occurred to a similar extent in the subgroup of patients with no or limited inflammation (SJC28 and CRP ≤ 1) compared with the rest of the cohort (Fig. 3A). The proportions of meaningful improvements and of pain relief (≤ 20 mm and ≤ 10) were again overall comparable, with a non-significant trend for deeper pain control in apparent pauci/non-inflammatory patients (Fig. 3B, C). Among them, proportions experiencing ≥ 70% pain improvement and residual pain ≤ 10 mm were indeed 40.4% and 35.7%, compared with 27.7% and 23.2% in the subgroup with overt inflammatory disease activity (*p* = 0.20 for ≥ 70% pain improvement; *p* = 0.14 for residual pain).

## Discussion

In this study, we provide evidence of the real-life effectiveness of JAKis in patients with RA in whom joint inflammation and painful symptoms were not controlled by the first or subsequent lines of therapy. We show that, beyond numerically significant reductions in pain scores, most patients experienced clinically meaningful improvements, with negligible residual pain in as many as 40% of the cases. Importantly, good pain outcomes were also achieved in patients escalated to treatment because of persistent pain in spite of limited discernible inflammation.

The clinical effectiveness of JAKis on different components of disease activity and impact in moderate-to-severe RA is well established in RCTs [18]. Most patients seen in routine care are, however, below the thresholds of disease activity required to enrol in these trials because of fewer swollen joints and systemic inflammation [19], and a significant yet undetermined proportion even lacks genuine inflammation despite being highly symptomatic [5, 6]. With the increasing availability of different treatments options, and the promising results of JAKis on pain, it is expected that more patients in various disease activity states are offered the opportunity to escalate therapies. The actual benefits of targeted agents in improving disease burden across the spectrum of ‘active’ RA remain, however, unexplored. Even the largest real-world experiences, such as the ‘JAK-pot’ collaboration, do not report specific outcomes based on different thresholds and domains of disease activity at treatment start [20]. Patients receiving JAKis at our division were representative of a mixed pattern of disease states, with the majority with moderately active RA, and a significant proportion of more than 30% apparently pauci-inflammatory. Notwithstanding such remarkably lower severity compared to RCTs, clinical outcomes were favourable, with 25–30% of the patients attaining stringent remission even in relation with previous bDMARDs exposure, in line with larger real-life populations [20]. Of note, the achievement of CDAI and Boolean remission in those patients already within the remission thresholds of swollen joints and acute phase implies improvement in the subjective domains of symptoms and self-perception. Accordingly, pain significantly decreased irrespective of baseline disease activity, and 50% of the patients with SJC28 and CRP ≤ 1 at treatment start achieved thresholds of mild pain, with one-third experiencing complete abatement of painful symptoms. The more favourable outcomes were observed in the less treatment-experienced patients, confirming that patient-reported outcomes have closer correlation with inflammation in the earliest stages of disease [21, 22].

Strengths of our study include the description of a prospective cohort reflecting the complexities of RA in clinical practice, with a significant number of patients deemed eligible to treatment escalation in spite of limited objective inflammation. Furthermore, rather than analysing changes in pain levels in mere numerical terms, we focused on outcomes that are clinically relevant to the patient. We acknowledge that our definition of pauci-inflammatory disease is arbitrary, and that the absence of clinically swollen joints does not exclude residual levels of subclinical inflammation. However, when the thresholds of stringent remission are satisfied, as in the case of SJC and CRP ≤ 1, the probability of ultrasound-detectable synovitis is low [23]. Our analysis focused on short-term outcomes, and a control group receiving a different biological agent was not included. Although RCTs have shown that pain control achieved with JAKis is durable and more significant than that obtained with other drugs [13–18, 24], confirmation in real-life settings is still pending. Also, paucity of head-to-head trials in RA [26] hampers comparison with other drugs targeting single cytokines involved in pain, such as IL-6 [27, 28]. Indication to treatment with JAKis in our study was antecedent to the restriction measures endorsed by regulatory agencies, so that the relatively high number of patients naive to other mechanisms of action hampers to define at which stage of the disease (and in which patients) pain eventually disconnects from inflammation [21, 22] and becomes mostly unresponsive to immunosuppressive treatment. However, RCTs in multi-refractory RA and experimental evidences indicate that the effect of JAKis on pain could be drug-specific [12, 25]. Last, though certainly of great importance, when assessing pain reduction as an outcome measure in open-label non-controlled observational studies, we cannot exclude placebo effects arising from patients' positive expectancies from novel treatments [29]. In our study, this might have been particularly the case of subjects escalated to JAKis immediately after failure of csDMARDs. However, we could demonstrate significant improvements in subjective pain also after failure of multiple lines of different treatments, as nearly 30% of the patients already exposed to ≥ 2 previous bDMARDs reported mild pain levels at the end of follow-up. Furthermore, RCTs of most the available JAKis in bDMARD-naïve patients have demonstrated significantly greater and/or faster control of patient-reported outcomes vs active comparator [15–17]. In conclusion, results of our study confirm the clinically significant and robust efficacy of JAKis on several domains of disease activity in RA, including pain, in routine practice. The observed improvement of painful symptoms also in those patients with doubtful inflammation may open new perspectives on the personalized management of difficult-to-treat RA.

## Data Availability

Data relevant to the study are included in the article. De-identified participant rough data are available from the corresponding author (serena.bugatti@unipv.it) upon reasonable request.

## Declarations

### Patient and public involvement

Patients and/or the public were not involved in the design, or conduct, or reporting, or dissemination plans of this research.
